# Diagnostic Approach to a Patient With Paraneoplastic Neurological Syndrome

**DOI:** 10.4021/wjon571w

**Published:** 2012-10-28

**Authors:** Ali Mahta, Namrata Vijayvergia, Tapan M. Bhavsar, Lawrence D. Ward

**Affiliations:** a Department of Medicine, Temple University Hospital, USA; b Department of Neurology, Temple University Hospital, USA; c Department of Pathology, Temple University Hospital, USA

**Keywords:** Paraneoplastic, Small cell lung cancer ataxia, Cerebellar degeneration

## Abstract

Herein, we discussed a case of an otherwise healthy man who presented with progressive gait imbalance and ataxia, found to have small cell lung cancer. Based upon our clinical findings and laboratory data, a diagnosis of paraneoplastic cerebellar degeneration was made. Paraneoplastic neurological syndromes (PNS) are relatively rare but diverse and always should be considered in differentials. A diagnostic algorithm along with appropriate work up is discussed here.

## Introduction

**Figure 1:**
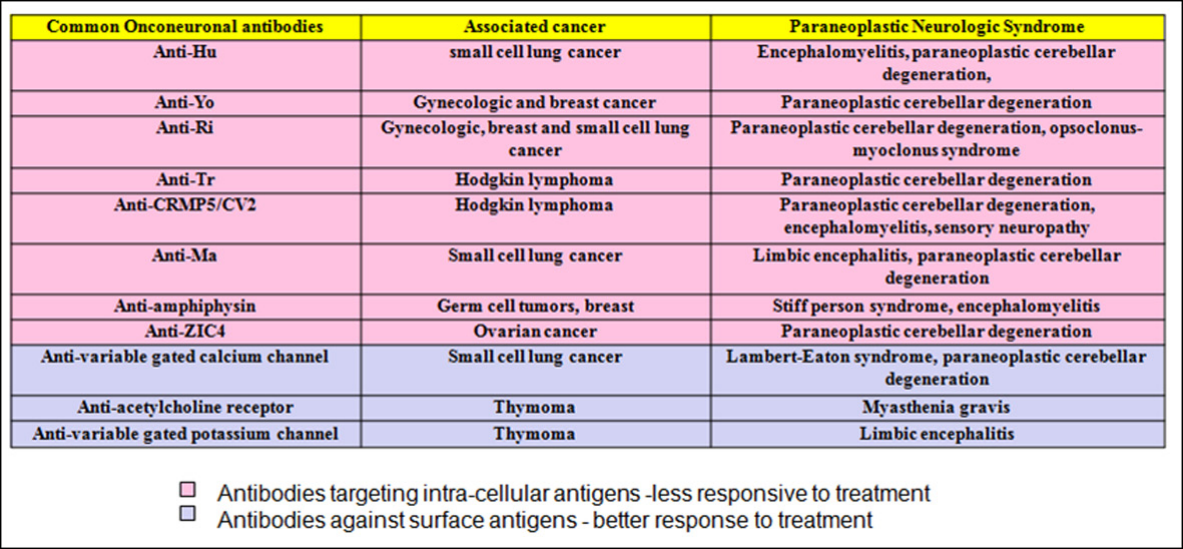
Common onconeuronal antibodies with their corresponding malignancies and paraneoplastic neurological syndromes.

Paraneoplastic neurological syndromes (PNS) are defined as any neurological dysfunction in a cancer patient in the absence of direct mass effect of the primary tumor or metastatic involvement of the central nervous system and it is caused by mechanisms other than metabolic or nutritional deficits, infections or coagulopathy or side effects of cancer treatment. The diagnosis should be established after excluding other etiologies such as infections or collagen vascular disorders. These paraneoplastic neurological syndromes are heterogeneous and caused by an immune response to an underlying malignancy in less than 1% of cancer patients [1]. This immune response is mediated by onconeural antibodies produced by tumor cells with some cross reactivity with components of the nervous system (Fig. 1). In addition to onconeuronal antibodies, cytotoxic T cells contribute to pathogenesis of paraneoplastic cerebellar degeneration [2].

## Case Report

**Figure 2:**
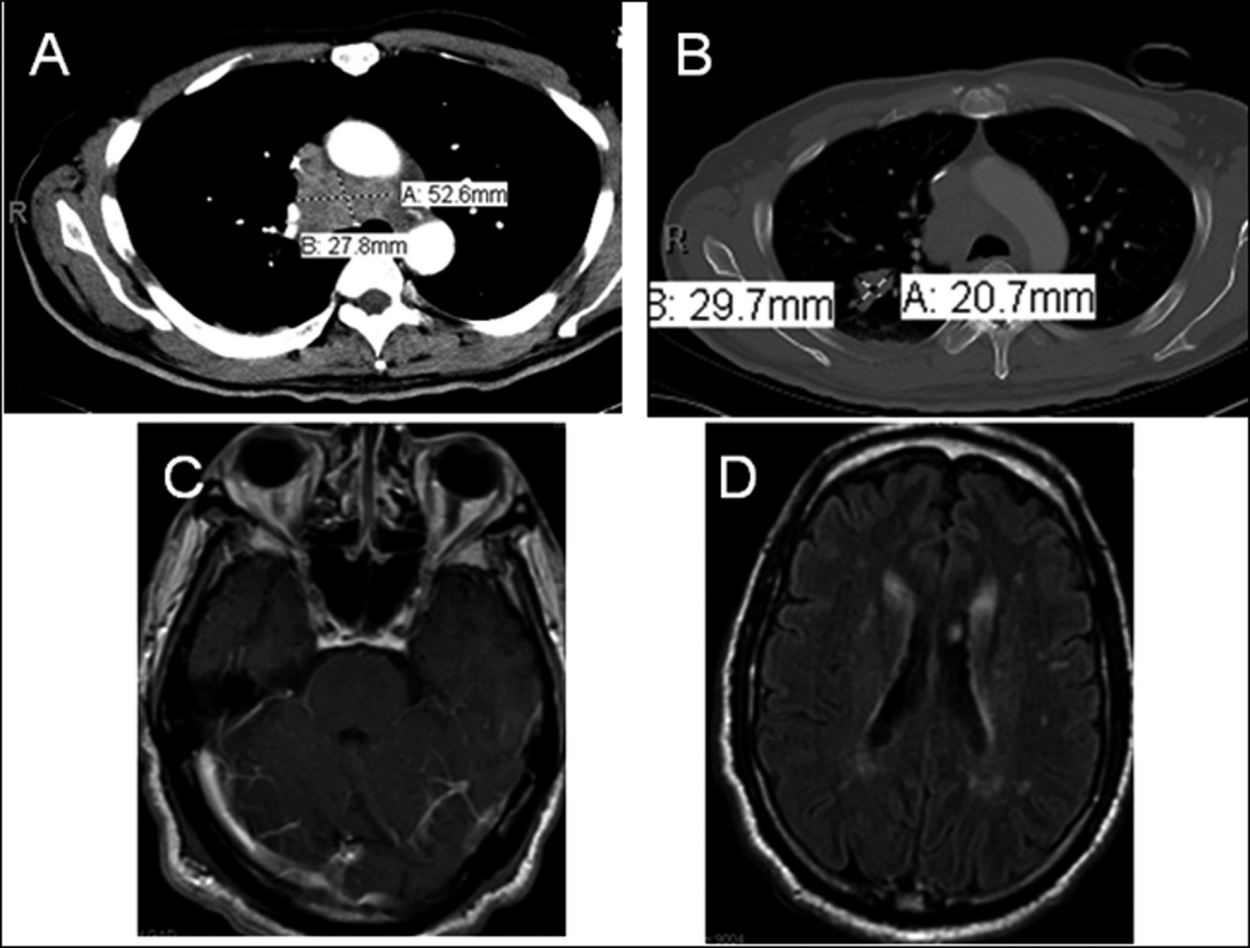
CT Chest demonstrating mediastinal mass (A) and right lung parenchymal lesion (B). Brain MRI T1 post-gad (C) showing cerebellar enhancement, FLAIR image (D) indicating diffuse white matter signal changes.

**Figure 3:**
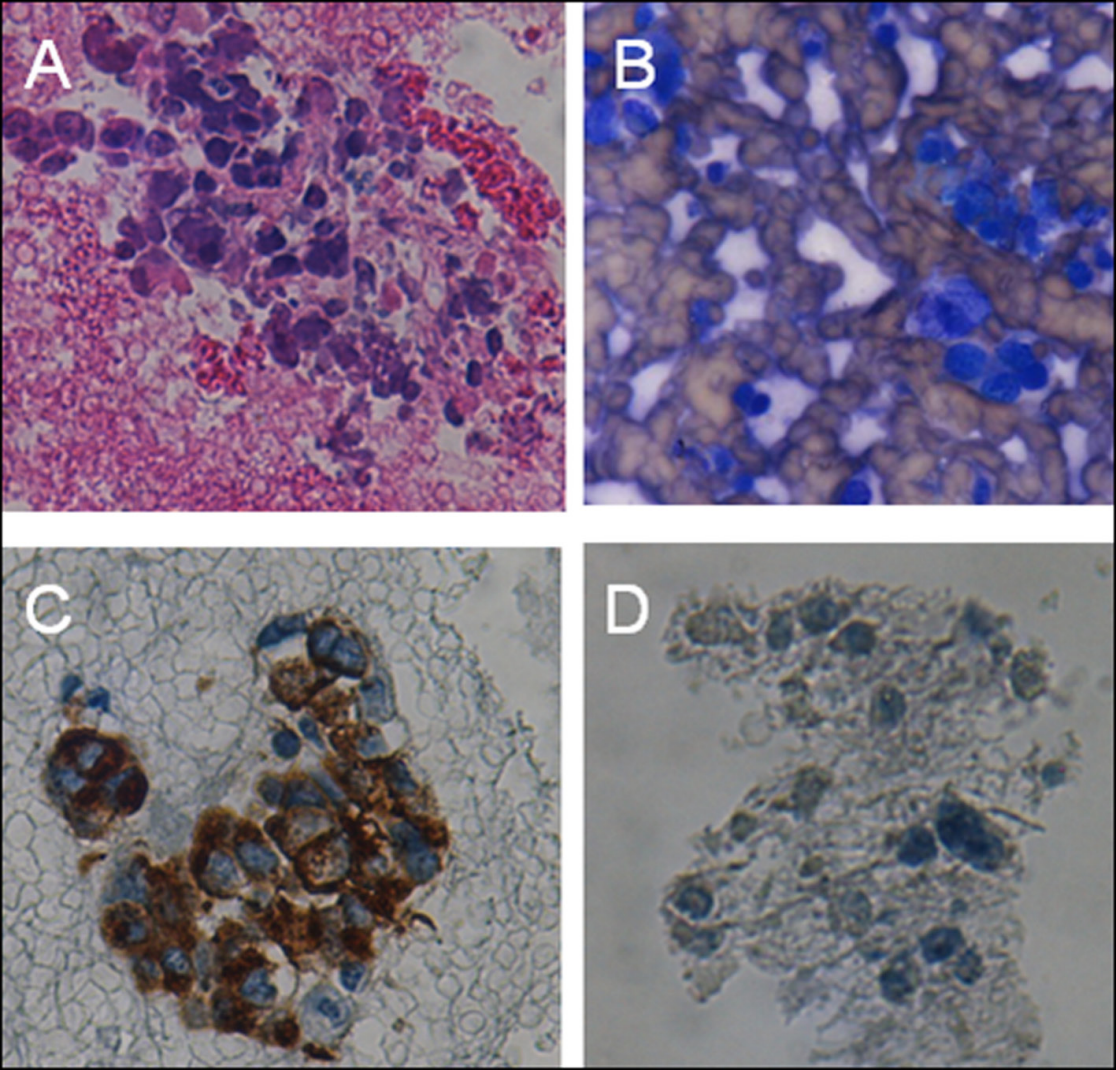
CT guided FNA from mediastinal mass showing tumoral cells (A-H&E stain, B-diff quick), Transbronchial biopsy of right lung lesion demonstrating small round cells with neuroendocrine features (C-Synaptophysin, D-Chromogranin stains).

A 64 year-old male with no significant past medical history other than hypertension presented with progressive gait imbalance and slurred speech over a period of 2 - 3 weeks. He denied any history of recent travel outside of urban Philadelphia, any sick contact or other constitutional symptoms. His social history was significant for smoking of one pack of cigarettes per day for about 30 years. He denied any history of hereditary neurodegenerative disorders in his family. Significant findings on neurologic exam included a severe gait imbalance, truncal ataxia, and dysarthria. A Brain MRI demonstrated a prominent enhancement along the inferior surface of the cerebellum along with diffuse white matter changes. A CT scan of the chest showed a large mass lesion involving the right upper lobe with mediastinal extension (Fig. 2). The patient underwent a CT guided fine needle aspiration (FNA) from the mediastinal mass, and transbronchial biopsy of the right lung lesion. The pathology was consistent with small cell lung cancer with endocrine features (Fig. 3).

A lumbar puncture was performed to rule out leptomeningeal carcinomatosis. Cerebrospinal fluid (CSF) analysis demonstrated WBC 5, RBC 300, protein 85 mg/dL and glucose 60 mg/dL. It was not remarkable for any viral encephalitides such as Herpes or west Nile and CSF cytology was also negative for any atypical or malignant cells. The patient underwent a brain and dural biopsy to rule our leptomeningeal carcinomatosis. We performed biopsy instead of less invasive methods like MR spectroscopy because MRI findings like leptomeningeal enhancement was concerning for direct neoplastic seeding. In addition, other radiographic findings like white matter changes which are less typical seen in a paraneoplastic syndrome, made us to proceed a brain biopsy. The pathology was negative for any metastatic lesion or any leptomeningeal involvement. It showed normal cortical grey and white matter cells with no significant pathologic changes.

Extensive laboratory work up was performed to rule out collagen vascular disorders or any possible infectious or metabolic etiologies. Based on history and clinical findings, our impression was paraneoplastic cerebellar degeneration due to the small cell lung cancer. Among onconeuronal antibodies, CSF anti-Hu antibody came back positive which confirms our diagnosis. We thought the white matter changes found on brain MRI were nonspecific and most likely caused by chronic small vessel changes due to long standing hypertension.

Systemic chemotherapy and plasmapheresis were initiated immediately. However; the patient rapidly deteriorated and became encephalopathic. The family decided to discontinue any further medical treatment based on his will prior to his altered mental status, hence he was discharged to a hospice care facility afterwards**.**

## Discussion

As it was evident by our patient, PNS usually precedes the diagnosis of cancer in 50 to 80% of cases. PNS can be arbitrarily categorized into 2 main groups: 1) classical syndromes including subacute cerebellar degeneration, limbic encephalitis, opsoclonus-myoclonus syndrome, Lambert-Eaton syndrome, encephalomyelitis, and 2) non-classical syndromes such as stiff person syndrome, optic neuritis, brain stem encephalitis and Myasthenia Gravis [1-4].

Small cell lung cancer has been notorious for causing PNS. The most common syndromes associated with small cell lung cancer include subacute cerebellar degeneration, brain stem and limbic encephalitides, opsoclonus-myoclonus syndrome, myelopathy, acquired neuromyotonia and Eaton-Lambert syndrome [3, 4].

**Figure 4:**
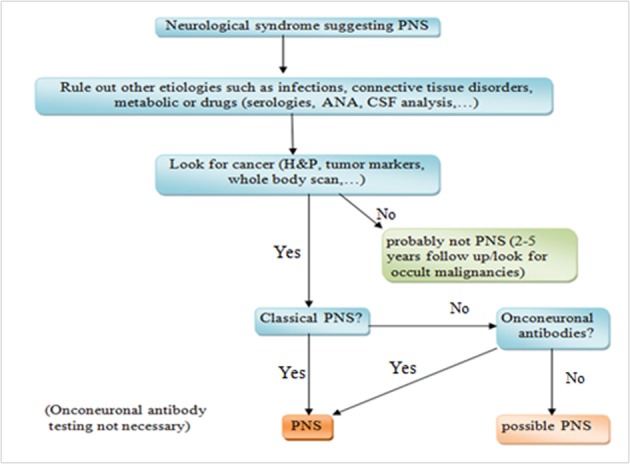
Diagnostic algorithm for how to approach to a patient with paraneoplastic neurological syndrome.

The first step in diagnosis of PNS is to rule out other etiologies including infections, connective tissue disorders, metabolic causes or even drug side effects. If the initial work up is inconclusive, the next step would be looking for malignancy based on history, physical exam and paraclinic findings. If there is an evidence of cancer and the neurological syndrome is “typical”, the diagnosis is highly suggestive of PNS and further testing for onconeuronal antibodies can be helpful but is not necessary. However; if the syndrome is not typical, onconeuronal antibodies should be tested (Fig. 4). If there is no evidence of cancer, it is still recommended a close follow up for up to 5 years in case we are dealing with an occult malignancy such as early stages of breast cancer.

Chemotherapy plus an immunosuppressive therapy and/or plasmapheresis should be initiated as soon as the diagnosis is established, however; the neurologic symptoms may or may not improve after treatment. Sometimes the type of antibody is helpful as a predictor for response to immunotherapy. As a general rule, tumors which produce antibodies targeting extra-cellular antigens such as acetyl choline receptor are more responsive to immunomodulatory therapies in contrast to tumors associated with antibodies against intracellular components such as anti-Hu and anti-MA (Fig. 1). In fact, lack of response to chemotherapy/immunosuppressive therapy does not rule out the diagnosis of PNS [1, 5, 6].
